# CloudSEN12, a global dataset for semantic understanding of cloud and cloud shadow in Sentinel-2

**DOI:** 10.1038/s41597-022-01878-2

**Published:** 2022-12-24

**Authors:** Cesar Aybar, Luis Ysuhuaylas, Jhomira Loja, Karen Gonzales, Fernando Herrera, Lesly Bautista, Roy Yali, Angie Flores, Lissette Diaz, Nicole Cuenca, Wendy Espinoza, Fernando Prudencio, Valeria Llactayo, David Montero, Martin Sudmanns, Dirk Tiede, Gonzalo Mateo-García, Luis Gómez-Chova

**Affiliations:** 1 Image Processing Laboratory, University of Valencia, 46980 Valencia, Spain; 2 Department of Geoinformatics – Z_GIS, University of Salzburg, 5020 Salzburg, Austria; 3 High Mountain Ecosystem Research Group, National University of San Marcos, 15081 Lima, Peru; 4 Research Group on Artificial Intelligence, Pontifical Catholic University of Peru, 15088 Lima, Peru; 5 Sub-directorate of Atmospheric and Hydrospheric Sciences, Geophysical Institute of Peru, 15012 Lima, Peru; 6 Remote Sensing Centre for Earth Systems Research (RSC4Earth), Leipzig University, 04103 Leipzig, Germany

**Keywords:** Geodynamics, Atmospheric dynamics, Atmospheric dynamics

## Abstract

Accurately characterizing clouds and their shadows is a long-standing problem in the Earth Observation community. Recent works showcase the necessity to improve cloud detection methods for imagery acquired by the Sentinel-2 satellites. However, the lack of consensus and transparency in existing reference datasets hampers the benchmarking of current cloud detection methods. Exploiting the analysis-ready data offered by the Copernicus program, we created CloudSEN12, a new multi-temporal global dataset to foster research in cloud and cloud shadow detection. CloudSEN12 has 49,400 image patches, including (1) Sentinel-2 level-1C and level-2A multi-spectral data, (2) Sentinel-1 synthetic aperture radar data, (3) auxiliary remote sensing products, (4) different hand-crafted annotations to label the presence of thick and thin clouds and cloud shadows, and (5) the results from eight state-of-the-art cloud detection algorithms. At present, CloudSEN12 exceeds all previous efforts in terms of annotation richness, scene variability, geographic distribution, metadata complexity, quality control, and number of samples.

## Abstract (summary)

**Table:** 

Measurement(s)	clouds extension
Technology Type(s)	machine learning with photo-interpretation.
Factor Type(s)	Copernicus Sentinel-2 images
Sample Characteristic - Environment	geosphere
Sample Characteristic - Location	all the world

## Background & Summary

We are in the midst of an exciting new era of Earth observation (EO), wherein Analysis Ready Data (ARD)^1–3^ products derived from big optical satellite imagery catalogs permit direct analyses without laborious pre-processing. Unfortunately, many of these products are contaminated by clouds^4^ and their corresponding shadows, altering the surface reflectance values and hampering their operational exploitation at large scales. For most of the applications exploiting ARD, cloud and cloud-shadow pixels need to be removed prior to further analyses, i.e. masked out, to avoid distortions in the results.

Improving the accuracy of existing cloud detection (CD) algorithms used in current ARD products is a pressing need for the EO community regarding optical sensors such as Sentinel-2. Ideally, CD algorithms would classify pixels into clear, cloud shadow, thin cloud, and thick cloud. Splitting clouds into two subclasses allows downstream applications to design different strategies to treat cloud contamination. On the one hand, thick clouds entirely block the surface’s view, reflecting most of the light coming from the sun and generating gaps impossible to retrieve using optical sensors data^5^. On the other hand, thin clouds do not reflect all the sunlight allowing to observe a distorted view of the surface^6,7^. For some applications, such as object detection or disaster response^8^, images contaminated with thin clouds are still helpful. Therefore, distinguishing between thick and thin clouds is also a critical first step toward optical data exploitation. Nevertheless, it is worth noting that there is no overall consensus on quantitative approaches delimiting when one class begins and the other ends; thus, it is so far inherently subjective to the image interpreter^9,10^.

Methodologies for CD can be classified into two main categories: knowledge-driven (KD) and data-driven (DD). KD category emphasizes the logical sense connected with physical foundations. For instance, the Function of mask (Fmask)^11^ and Sen2Cor^12^ use a set of physical rules formulated on spectral and contextual features to distinguish clouds against water or land. Overall, KD algorithms achieve accurate results, and good generalization^13–15^. However, it is well-known that they have problems associated with thin cloud omission and non-cloud object commission, frequently at cloud edges and under surfaces with a smooth texture or high reflectance^16,17^.

In recent years, supervised data-driven strategies, trained in large manually annotated datasets, have grown notoriety in remote sensing thanks to the success of classical machine learning (ML) and deep learning (DL) techniques^18^. Among multiple noteworthy ML precedents^19–21^ in cloud detection, Sentinel Hub’s s2cloudless^22^ is the most extensively used due to its low computational requirements and lightweight design. Nonetheless, when evaluated in certain particular regions, such as tropical forests, s2cloudless falls short of *state-of-the-art* KD cloud detectors^13,23,24^. Meanwhile, DL has proven to be more effective on CD compared to more classical ML^25,26^, although it is subjected to the exigency of pixel-level annotation.

The recent progress in DL-based cloud semantic segmentation in Sentinel-2 can be attributed to the proliferation of public CD datasets such as SPARCS^27^, S2-Hollstein^28^, Biome 8^10^, 38-cloud^29^, CESBIO^30^, 95-Cloud^31^, and CloudCatalogue^32^. Nonetheless, these datasets have some well-known shortcomings, including the absence of a temporal component, a lack of thin clouds or cloud shadows labels, a high degree of imbalance between cloud and non-cloud classes, and a relatively small size joined with geographical bias (see Table 1 for the current characteristics/limitations of each of those datasets). Furthermore, their quality control process is not always properly described and their development remains somehow unclear. Additionally, there is a lack of consensus on manual annotation protocols and cloud semantic classes definition, which makes inter-dataset comparison problematic, particularly under pixels where class transitions occur. These flaws hinder the natural transition to global DL cloud classifiers and the application of new-fashioned geographically-aware algorithms^13^.

**Table 1:** Summary of publicly available CD datasets in comparison to CloudSEN12. An asterisk represents that the dataset does not distinguish the specific class.

Name	Main region	Labels	# of Scenes	Temporal	# of Pixels (10^9^)	Thick Clouds%	Thin Clouds%	Cloud Shadows%	Clear%
L8-SPARCS^79^	worldwide	full-scene	80	No	0.080	19.37	*	7.37	73.26
S2-Hollstein^28^	Europe	polygons	59	No	0.003	16.06	16.49	4.53	62.92
L8-Biome8^10^	worldwide	full-scene	96	No	3.964	33.19	14.71	1.55	50.55
L8-38Cloud^29^	USA	full-scene	38	No	1.494	52.36	*	*	47.64
S2-CESBIO^30^	Europe	full-scene	38	No	0.109	22.77	*	2.71	74.52
L8-95Cloud^31^	USA	full-scene	95	No	3.737	49.27	*	*	50.73
S2-cloudCatalog^32^	worldwide	partial scene	513	No	0.535	52.58	*	1.47	45.95
WHUS2-CD^80^	China	full-scene	32	No	4.273	13.50	*	*	86.50
KappaZeta^54^	Northern Europe	partial scene	155	No	1.064	34.37	19.21	8.36	38.05
**CloudSEN12**	**worldwide**	**partial scene**	**46697**	**Yes**	**4.697**	**15.50**	**5.52**	**5.24**	**73.73**

**Fig. 1:**
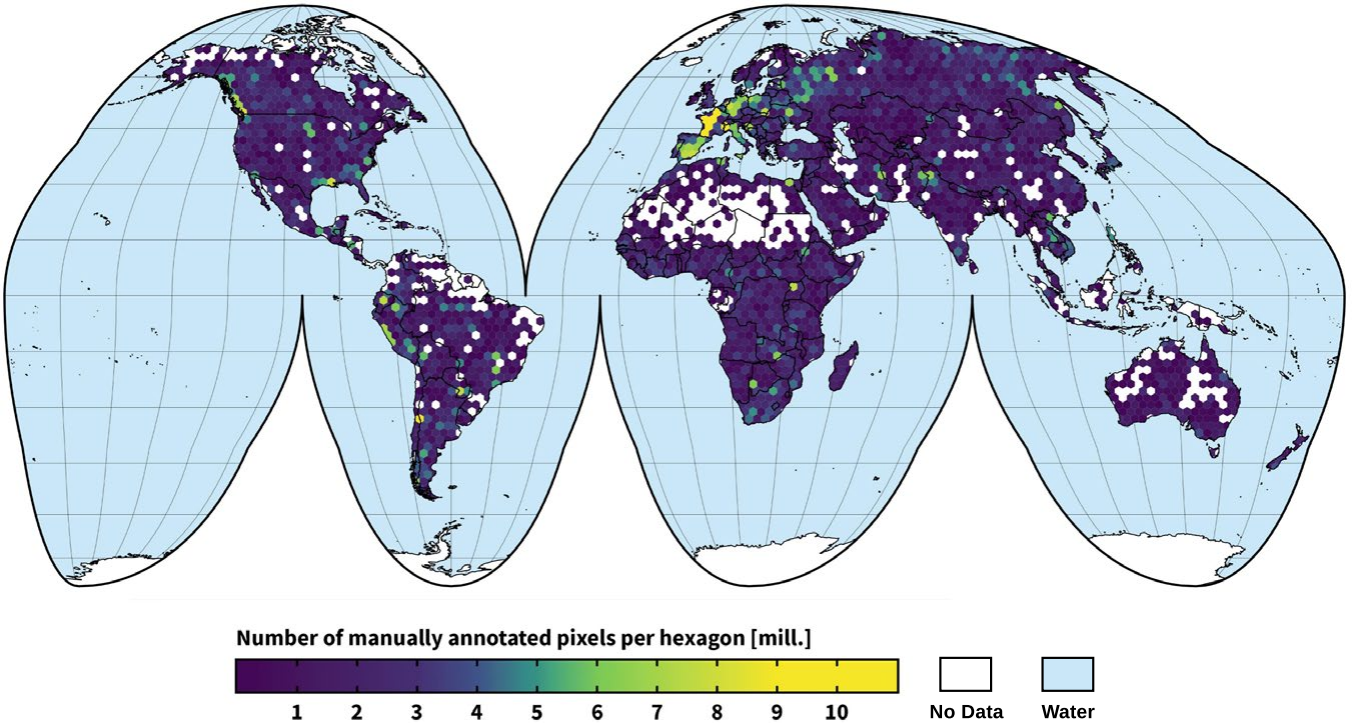
CloudSEN12 spatial coverage, purple-to-yellow color gradient represents the amount of manually annotated pixels per hexagon. The annotated pixels were collocated in an equal-area hexagonal discrete grid with a facet size of 140 km.

Inspired by the CityScapes dataset^33^, we created and released CloudSEN12, a large and globally distributed dataset (Fig. 1) for cloud semantic understanding based mainly on Sentinel 2 imagery. CloudSEN12 surpasses all previous efforts in size and variability (see Table 1), offering 49,250 image patches (IPs) with different annotation types: (i) 10,000 IPs with high-quality pixel-level annotation, (ii) 10,000 IPs with scribble annotation, and (iii) 29,250 unlabeled IPs. The labeling phase was conducted by 14 domain experts using a supervised active learning system. We designed a rigorous four-step quality control protocol based on Zhu *et al*.^34^ to guarantee high quality in the manual annotation phase. Furthermore, CloudSEN12 ensures that for the same geographical location, users can obtain multiple IPs with different cloud coverage: cloud-free (0%), almost-clear (0–25%), low-cloudy (25–45%), mid-cloudy (45–65%), and cloudy (>65%), which ensures scene variability in the temporal domain. Finally, to support multi-modal cloud removal^35^ and data fusion^36^ approaches, each CloudSEN12 IP includes data from various remote sensing sources that have already shown their usefulness in cloud and cloud shadow masking, such as Sentinel-1 and elevation data. See Table 2 for a full list of assets available for each image patch.

**Table 2:** List of assets available for each image patch.

File/Folder	Name	Scale	Wavelength	Description
S2L1C & S2L2A	B1	0.0001	443.9 nm (S2A)/442.3 nm (S2B)	Aerosols.
	B2	0.0001	496.6 nm (S2A)/492.1 nm (S2B)	Blue.
	B3	0.0001	560 nm (S2A)/559 nm (S2B)	Green.
	B4	0.0001	664.5 nm (S2A)/665 nm (S2B)	Red.
	B5	0.0001	703.9 nm (S2A)/703.8 nm (S2B)	Red Edge 1.
	B6	0.0001	740.2 nm (S2A)/739.1 nm (S2B)	Red Edge 2.
	B7	0.0001	782.5 nm (S2A)/779.7 nm (S2B)	Red Edge 3.
	B8	0.0001	835.1 nm (S2A)/833 nm (S2B)	NIR.
	B8A	0.0001	864.8 nm (S2A)/864 nm (S2B)	Red Edge 4.
	B9	0.0001	945 nm (S2A)/943.2 nm (S2B)	Water vapor.
	B11	0.0001	1613.7 nm (S2A)/1610.4 nm (S2B)	SWIR 1.
	B12	0.0001	2202.4 nm (S2A)/2185.7 nm (S2B)	SWIR 2.
S2L1C	B10	0.0001	1373.5 nm (S2A)/1376.9 nm (S2B)	Cirrus.
S2L2A	AOT	0.001	—	Aerosol Optical Thickness.
	WVP	0.001	—	Water Vapor Pressure.
	TCI_R	1	—	True Color Image, Red.
	TCI_G	1	—	True Color Image, Green.
	TCI_B	1	—	True Color Image, Blue.
S1	VV	1	5.405 GHz	Dual-band cross-polarization, vertical transmit/horizontal receive.
	VH	1	5.405 GHz	Single co-polarization, vertical transmit/vertical receive.
	angle	1	—	Incidence angle generated by interpolating the ‘incidenceAngle’ property.
extra/	CDI	0.0001	—	Cloud Displacement Index^41^.
	Shwdirection	0.01	—	Azimuth. Values range from 0°–360°
	elevation	1	—	Elevation in meters. Obtained from MERIT Hydro datasets^38^.
	ocurrence	1	—	JRC Global Surface Water^39^. The frequency with which water was present.
	LC100	1	—	Copernicus land cover product. CGLS-LC100 Collection 3^40^.
	LC10	1	—	ESA WorldCover 10 m v100 product.
labels/	fmask^11^	1	—	Fmask4.0 cloud masking.
	QA60	1	—	SEN2 Level-1C cloud mask.
	s2cloudless^22^	1	—	sen2cloudless results.
	sen2cor	1	—	Scene Classification band. Obtained from SEN2 level 2A.
	CD-FCNN-RGBI	1	—	López-Puigdollers *et al*.^23^ results based on RGBI bands.
	CD-FCNN-RGBISWIR	1	—	López-Puigdollers *et al*.^23^ results based on RGBISWIR bands.
	kappamask_L1C	1	—	KappaMask^54^ results using SEN2 level L1C as input.
	kappamask_L2A	1	—	KappaMask^54^ results using SEN2 level L2A as input.
	manual_hq	1		High-quality pixel-wise manual annotation.
	manual_sc	1		Scribble manual annotation.

## Methods

**Fig. 2:**
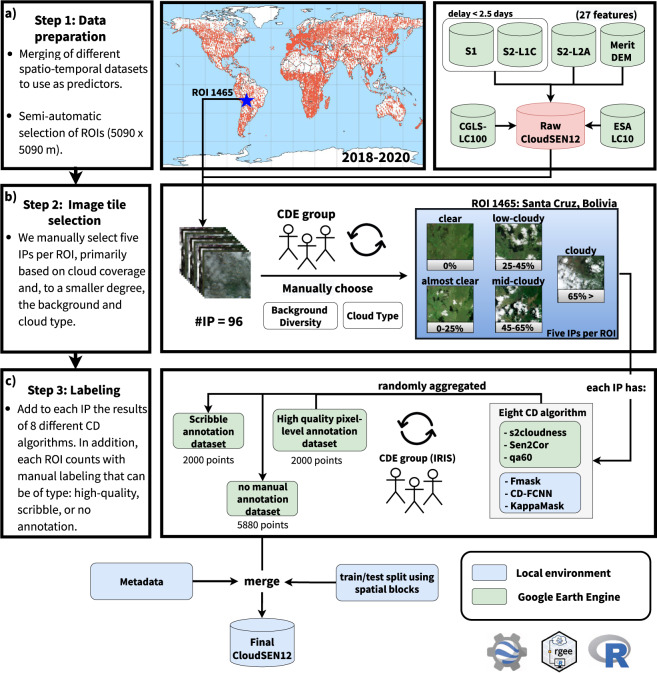
A high-level summary of our workflow to generate IPs. (**a**) Satellite imagery datasets that comprises CloudSEN12 assets. (**b**) IP selection by the CDE group. (**c**) Generation of manual and automatic cloud masking. KappasMask and CD-FCNN have two distinct configurations.

This study collects and combines several public data sources that may potentially help us to annotate cloud and cloud shadows better. Based on this information, semantic classes (Table 3) are created using an active system that blends human photo interpretation and machine learning. Finally, a strict quality control protocol is carried out to ensure the highest quality on the manual labels and to establish human-level performance. Figure 2 depicts the whole workflow followed to create the dataset. Figure 2a depicts all available data in each CloudSEN12 IP (see Method:Data preparation section). Figure 2b illustrates the manual IP selection strategy realized in each ROI (see Method:Image patches selection section). Finally, Fig. 2c highlights the human annotation strategy and cloud detection models offered in each IP (see Method:Annotation strategy and Method:Available cloud detection models sections).

**Table 3:** Cloud semantic categories considered in CloudSEN12. Lower priority levels indicate greater relevance.

Code	Class	Superclass 1	Superclass 2	Description	Priority
0	Clear	non-cloud	valid	Pixels without cloud and cloud shadow contamination.	4
1	Thick Cloud	cloud	invalid	Opaque clouds that block all the reflectance from the Earth’s surface.	1
2	Thin Cloud	cloud	invalid	Semitransparent cloud that alters the surface spectral signal but still allows to recognize the background.	3
3	Cloud Shadow	non-cloud	invalid	Dark pixels thrown by a thick or thin cloud.	2

### Data preparation

CloudSEN12 comprises different free and open datasets provided by several public institutions and made accessible by the Google Earth Engine (GEE) platform^37^. These include Sentinel-2A/B (SEN2), Sentinel-1A/B (SEN1), Multi-Error-Removed Improved-Terrain (MERIT) DEM^38^, Global Surface Water^39^ (GSW), and Global Land Cover maps^40^ at 10 and 100 meters. The SEN1 and SEN2 multi-spectral image data correspond to the 2018–2020 period. We included all the bands from both SEN2 top-of-atmosphere (TOA) reflectance (Level-1C) and SEN2 surface reflectance (SR) values (Level-2A) derived from the Sen2Cor processor, which can be useful to analyze the impact of CD algorithms on atmospherically corrected derived products. See *S2L1C* and *S2L2A* in Table 2 for band description. On the other hand, SEN1 acquires data with a revisit cycle between 6–12 days according to four standard operational modes: Stripmap (SM), Extra Wide Swath (EW), Wave (WV), and Interferometric Wide Swath (IW). In CloudSEN12, we collect IW data with two polarization channels (VV and VH) from the high-resolution Level-1 Ground Range Detected (GRD) product. Furthermore, we saved the approximate angle between the incident SAR beam and the reference ellipsoid (see *S1* in Table 2). Lastly, our dataset also includes previously proposed features for cloud semantic segmentation such as (1) Cloud Displacement Index^41^, (2) the azimuth (0–360°) calculated using the solar azimuth and zenith angles^42^ from SEN2 metadata, (3) elevation from MERIT dataset, (4) land cover maps from the Copernicus Global Land Service (CGLS) version 3, and the ESA WorldCover 10 m v100, and (5) water occurrence from the GSW dataset (see *extra/* in Table 2). All the previous features constitute the raw CloudSEN12 imagery dataset (Fig. 2a). All the image scenes in raw CloudSEN12 were resampled to 10 meters using local SEN2 UTM coordinates.

### Image patches selection

We sampled 20,000 random regions of interest (ROIs) dispersed globally in order to retrieve raw CloudSEN12 data. Each ROI has a dimension of 5,090 × 5,090 meters. Besides, we carefully added 5,000 manually selected ROIs to guarantee high scene diversity on complicated surfaces such as snow and built-up areas. Afterwards, an ROI is retained in the dataset if all three of the following requirements are met: (1) SEN2 Level-1C IP does not include saturated or no-data pixel values, (2) the time difference between SEN1 and SEN2 acquisitions is not higher than 2.5 days, and (3) there are more than 15 SEN2 Level-1C image scenes for the given ROI after applying (2). The total number of ROIs decreased from 25,000 to 12,121 as a result of this filtering. Despite this reduction, CloudSEN12 still manages to reach a full global representation. However, a high number of ROIs does not necessarily imply a consistent distribution among cloud types and coverage. Unfortunately, image selection based on automatic cloud masking or cloud cover metadata tends to produce misleading results, especially under high-altitude areas^43^, intricate backgrounds^44^, and mixed cloud types scenes. Hence, to guarantee unbiased distribution between clear, cloud and cloud shadow pixels, 14 cloud detection experts manually selected the IPs (hereafter referred to as CDE group, Fig. 2b). For each ROI, we pick five IPs with different cloud coverage: cloud-free (0%), almost-clear (0–25%), low-cloudy (25–65%), mid-cloudy (45–65%), and cloudy image (>65%). Atypical clouds such as contrails, ice clouds, and haze/fog had a higher priority than common clouds (i.e., cumulus and stratus). After eliminating ROIs that did not count with at least one IP for each cloud coverage class, the total number of ROIs was reduced from 12,121 to 9,880, resulting in the final CloudSEN12 spatial coverage (Fig. 1).

### Annotation strategy

**Fig. 3:**
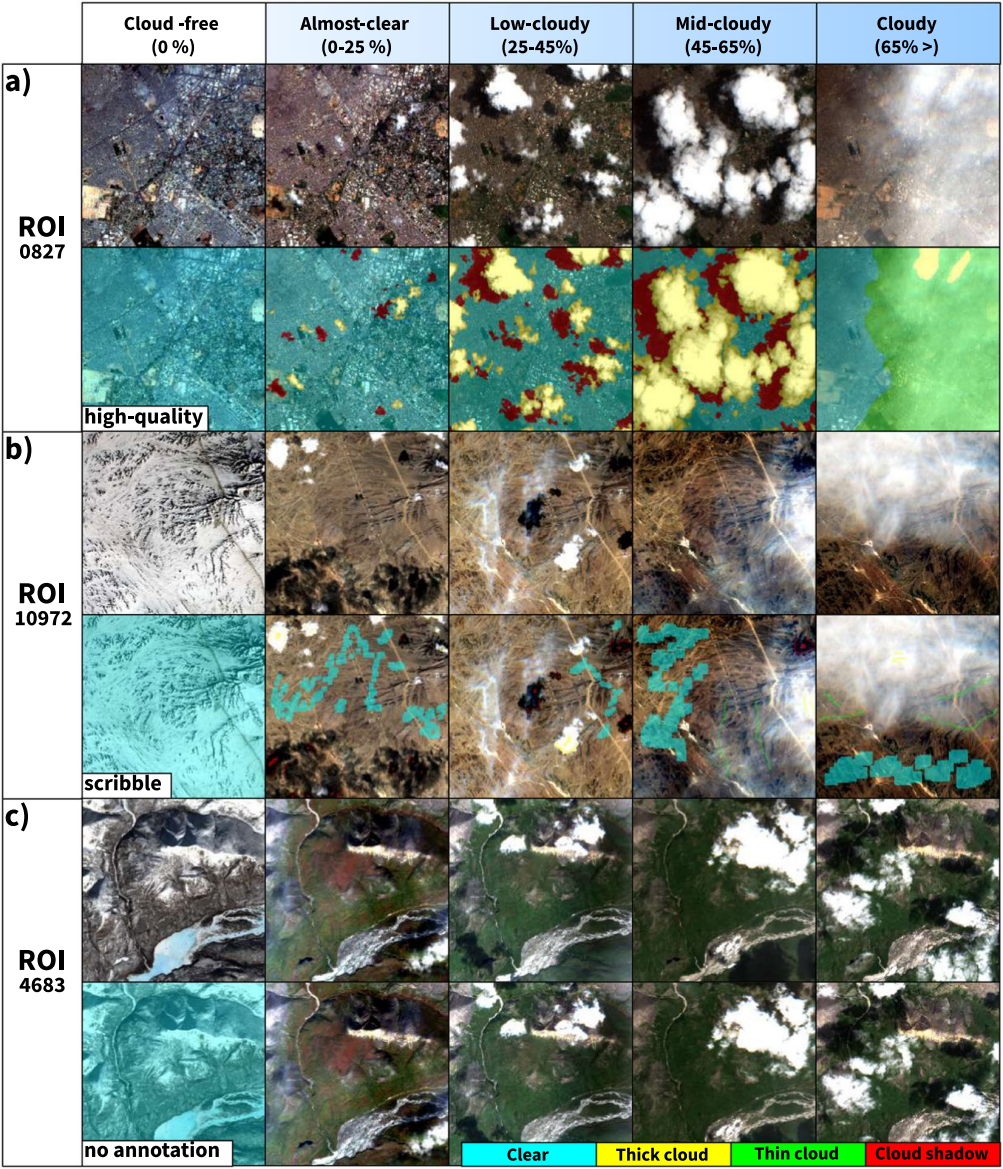
The three primary types of hand-crafted labeling data available in CloudSEN12. The first row in high-quality (**a**), scribble (**b**), and no annotation (**c**) subgroups shows a SEN2 level 1 C RGB band combination.
- 2,000 ROIs with scribble level annotation, where the annotation time is 16 minutes (scribble subset, Fig. 3b).
- 5,880 ROIs with annotation only in the cloud-free (0%) image (no annotation subset, Fig. 3c).

New trends in computer vision show that reformulating the standard supervised learning scheme can alleviate the huge demands of hand-crafted labeled data. For instance, semi-supervised learning can produce more detailed and uniform predictions^45^, while weakly-supervised learning suggests a more cost-effective option to pixel-wise annotation in semantic segmentation. Users might utilize scribble labels to train a model for coarse-to-fine enrichment^46^. Aware of these manual labeling requirements, CloudSEN12 includes three types of labeling data: high-quality, scribble, and no-annotation. Consequently, each ROI is randomly assigned to a distinct annotation category (Fig. 2c) and labelled by the CDE group:

- 2,000 ROIs with pixel level annotation, where the average annotation time is 150 minutes (high-quality subset, Fig. 3a).

### Human calibration phase

**Fig. 4:**
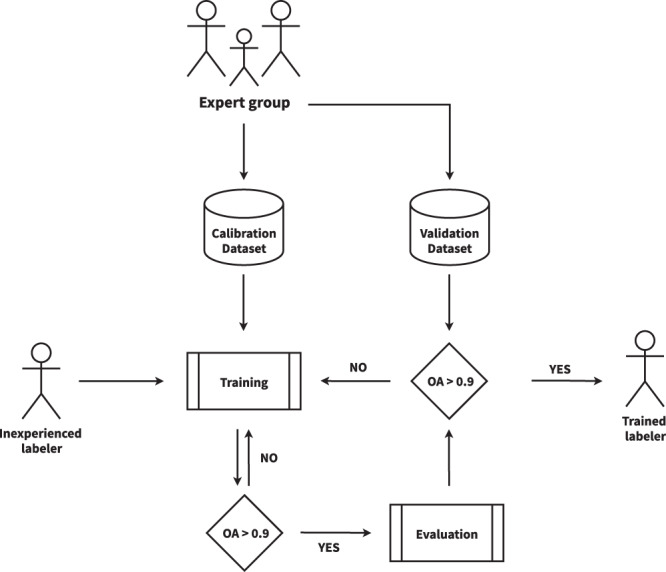
Human calibration workflow diagram. The overall accuracy (OA) is calculated by comparing individual labeler results against expert group results.

Human photo interpretation is not a faultless procedure. It might easily be skewed by an individual’s basis, overconfidence, tiredness, or ostrich-effect^47^ proclivity. Hence, to lessen this effect, the CDE group refined their criteria using a “calibration” dataset composed of 35 manually selected challenging IPs. In this stage, all the labelers can consult each other. As a result, they reached an agreement about the SEN2 band compositions to be used and how to deal with complicated scenarios such as cloud boundaries, thin cloud shadows, and high-reflectance background. A labeler is considered fully trained if its overall accuracy in the calibration dataset surpasses 90%. Then, a “validation” dataset formed of ten IPs is used to assess individual performance; labelers are not permitted to confer with one another during this step. If the labeler’s overall accuracy drops below 90%, it will return to the calibration phase (Fig. 4).

### Labeling phase

**Fig. 5:**
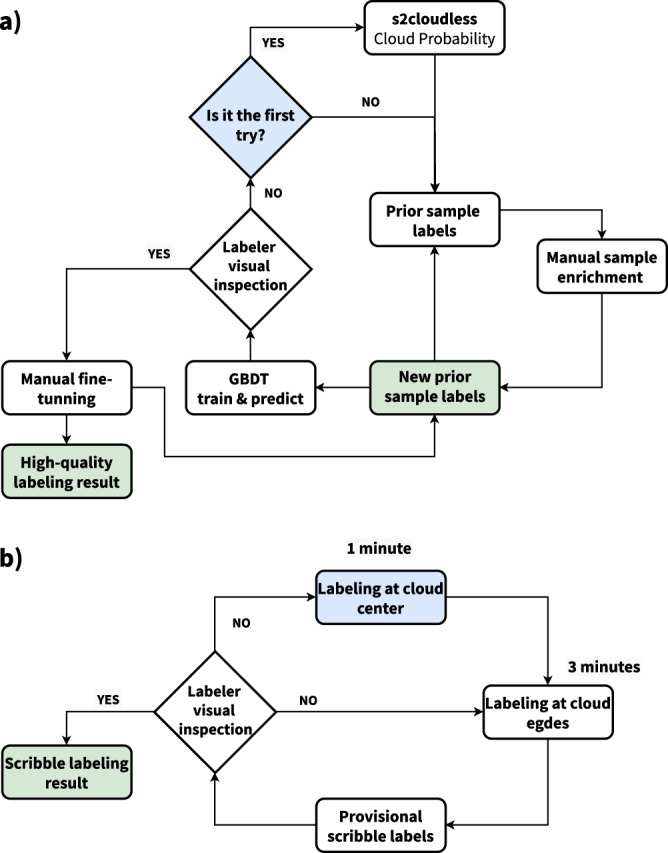
(**a**) High-quality labeling phase diagram. The model is set up using s2cloudless priors (blue). Annotations made by labelers with and without ML assistance are saved (green). (**b**) Scribble labeling phase diagram. The labelers starts by adding samples at the centroids (blue), and then into the borders; if the results pass a simple visual inspection, the annotation is send to inspection (see Method:Quality control phase section).

The Intelligence foR Image Segmentation (IRIS) active learning software^48^ was used in the manual labeling annotation process (Supplementary Fig. S1). IRIS allowed CDE members to train a model (learner) with a small set of labeled samples that is iteratively reinforced by acquiring new samples provided by a labeler (oracle). As a result, it dramatically decreases the time spent creating hand-crafted labels but maintains the labeler’s capacity to make final manual revisions if necessary. For high-quality labeling generation (Fig. 5a), IRIS starts training a gradient boosting decision tree (GBDT)^49^ with s2cloudless cloud probability values greater than 0.7 as thick cloud and less than 0.3 as clear. GBDT algorithm starts generating weak decision trees by dividing training data and gradually moving in the direction of lowering the loss function, then all the weak decision trees are combined into a single strong learner to generate a final prediction. IRIS use the LightGBM Python package. Readers are referred to Ke *et al*.^50^ for a detailed algorithm description. After obtaining GBDT predictions, the CDE group makes adjustments to the prior results and, if necessary, adds other cloud semantic classes such as cloud shadow and thin cloud. Using this new sample set, the GBDT model is re-trained. The two previous steps are repeated several times until the pixel-wise annotation passes the labeler’s visual inspection filter. The final high-quality annotation results are then obtained by applying extra manual fine-tuning. Since there are no quantitative criteria to distinguish between boundaries in semantic classes, the labelers always attempt to maximize the sensitivity score under ambiguous edges.

On the other hand, for scribble labeling (Fig. 5b), the CDE group also used IRIS but without ML assistance. First, labelers spend one-minute adding annotations around centroids of the semantic classes. Usually, pixels adjacent to the centroids are more straightforward to classify automatically. Then, to produce balanced annotations, the CDE group added more samples at cloud and cloud shadow edges for three more minutes.

### Quality control phase

**Fig. 6:**
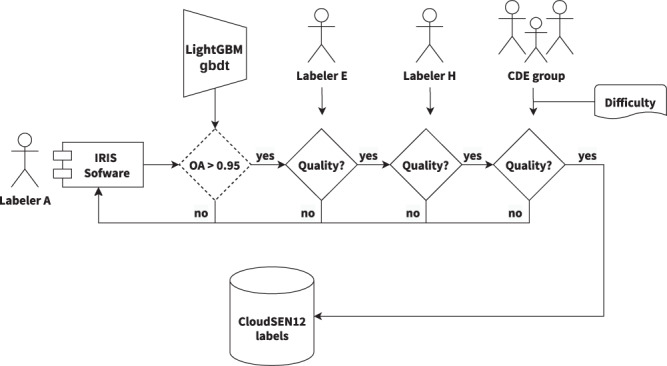
Flowchart overview of the entire QC process.

Despite the human calibration phase, errors are still common in hand-operated labels. Therefore, statistic and visual inspections were implemented before admitting a manual annotation in CloudSEN12 (Fig. 6). First, an automatic check is set only for high-quality labels. It proposes that the GBDT accuracy during training must be higher than 0.95. This simple threshold pushes the CDE group to set more samples and care more about labeling correctness. Later, two sequential visual inspection rounds are carried out for scribble and high-quality labels. The evaluators are two other CDE members than the one who labeled the IP. If a mistake is found, it is notified using GitHub Discussions (https://github.com/cloudsen12/models/discussions). Finally, we discern the most challenging IPs (difficulty level greater than 4, see Table 4) and consult all CDE members to reaffirm or change a semantic class. The deliberations were supported by using cloudApp (https://csaybar.users.earthengine.app/view/cloudapp), which is a GEE web application that displays SEN2 image time series from any location on the earth (Supplementary Fig. S2).

**Table 4:** Metadata associated to each image patch.

Metadata name	Description
annotator_name	The labeler’s name.
roi_id	The region of interest ID.
s2_id_gee	Sentinel-2 GEE ID.
s2_id	Sentinel-2 product ID.
s2_date	Sentinel-2 acquisition date in ISO format.
s2_sen2cor_version	Sen2Cor configuration baseline used at the time of the product generation.
s2_fmask_version	Fmask version.
s2_s2cloudless_version	s2cloudless version.
s2_reflectance_conversion_correction	Earth-Sun distance correction factor.
s2_aot_retrieval_accuracy	Accuracy of aerosol optical thickness model.
s2_water_vapour_retrieval_accuracy	Declared accuracy of the Water Vapor model.
s2_view_off_nadir	The angle from the SEN2 sensor between nadir (straight down) and the scene center.
s2_view_sun_azimuth	SEN2 sun azimuth angle.
s2_view_sun_elevation	SEN2 sun elevation angle.
s1_id	SEN1 product ID.
s1_date	SEN1 acquisition date in ISO format.
s1_grd_post_processing_software_name	Name of the software to pre-processing SEN1.
s1_grd_post_processing_software_version	SEN1 software pre-processing version.
s1_slc_processing_facility_name	Name of the facility where the processing step was performed.
s1_slc_processing_software_version	Software version identification.
s1_radar_coverage	percentage of valid SEN1 pixels contained in this IP.
land_cover	Predominant land use.
label_type	Manual labeling type (i.e., scribble, high-quality or no-annotation).
cloud_coverage	Cloud coverage estimated using photo-interpretation. (see Method: Image patches selection section).
test	Whether the IP is part of training (train) or testing (test) dataset.
difficulty	Labeler’s confidence (from 1 to 5) of the manual annotation.Where one indicates near-perfect and five denotes potentially significant mistakes.
proj:epsg	EPSG code.
proj:geometry	Footprint of this IP.
proj:shape	Number of pixels for the default IP.
proj:centroid	Centroid coordinates of the IP in latitude and longitude.
proj:transform	The affine transformation coefficients.

**Fig. 7:**
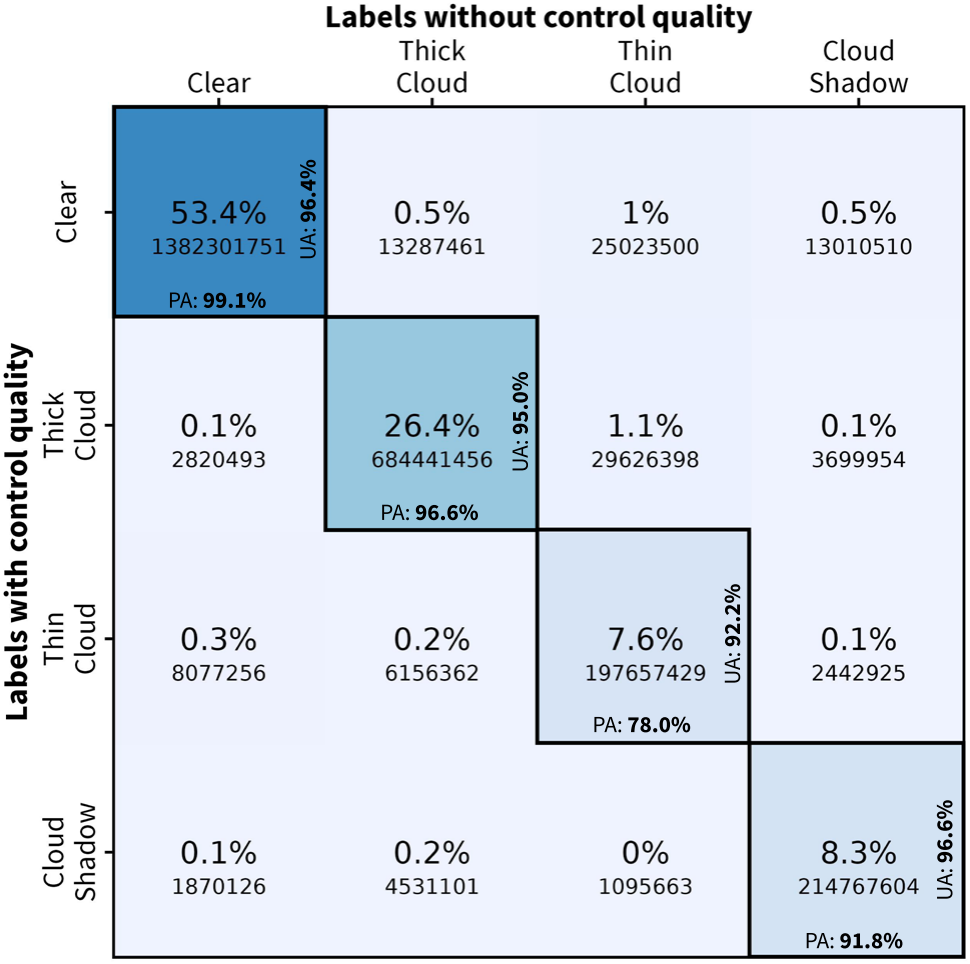
Confusion matrix between high-quality manual labels cast by the CDE group before and after the quality control procedure. In the middle of each tile, we show the number of pixels and their ratios with respect to the total number of pixels. The true positive class agreement is expressed by the UA and PA at the right and bottom of the diagonal tiles.

Comparing the manual annotation before and after quality control can provide insight into the correctness of annotations made by humans. Based on this, CloudSEN12 set, for the first time, the human-level performance at 95.7% confidence when considering all semantic cloud classes (described in Table 3) and 98.3% if thin clouds are discarded. The clear and thick cloud classes presented the largest PA agreement with 99.1% and 96.6%, respectively (Fig. 7). The variance concerning the thick cloud class (3.4%) was produced by efforts to limit the formation of false positives around cloud borders in the first round of quality control. In contrast, thin cloud and cloud shadow classes present the largest disparities, with a PA of 78 and 91.8%, respectively. Despite using IRIS and CloudApp, which permits labelers to contrast both spectral and temporal SEN2 data, the detection of semi-transparent clouds remained unclear (21%). This was especially noticeable when all CDE members discussed the most complicated IPs (Fig. 6); thin clouds were always the source of the most contention. Considering the assimilation of atmospheric reanalysis data and radiative transfer model outputs could help to reduce the cirrus detection uncertainty^9,51^. Nonetheless, our manual labeling approach did not consider this additional data. Finally, cloud shadow disagreement is explained by a reinterpretation of the semantic classes after the first quality control round. At first, we assumed that only thick clouds could project shadows. However, this was ruled out as many thin low-altitude clouds project their shadows on the surface, significantly affecting surface reflectance values.

### Available cloud detection models

The large number of user requirements makes challenging to compare CD algorithms fairly^24^. In crop detection, for instance, examining the performance of CD models during specific seasons rather than on an interannual scale may be more meaningful. Another example is that some data users may want to compare CD model performance geographically across different biomes or land-cover classes. In EO research that uses deep learning, it has become rather common to benchmark models as classic computer vision algorithms, generating global metric values for each validation dataset. However, this convenient approach is more likely to result in biased conclusions, especially using poorly distributed datasets. We argue that an appropriate model in EO must be capable of obtaining adequate global metrics while being consistent in space across multiple timescales, i.e., at the local domain. Furthermore, in cloud detection, the observed patterns must be aligned with our physical understanding of the phenomena. All of the above is hard to express in a single global metric value. Therefore, in order to cover all the possible EO benchmarking user requirements, we added to each IP the results of eight of the most popular CD algorithms (see *labels/*in Table 2). This simple step provides CloudSEN12 users more flexibility to choose a better comparison strategy tailored to their requirements. Next, we detail the CD algorithms available for each IP in CloudSEN12:

- Fmask4: Function of Mask cloud detection algorithm for Landsat and Sentinel-2^11^. We use the authors’ MATLAB implementation code via Linux Docker containers (https://github.com/cloudsen12/models). We set the dilatation parameter for cloud, cloud shadow, and snow to 3, 3, and 0 pixels, respectively. The erosion radius (dilation) is set to 0 (90) meters, while the cloud probability threshold is fixed to 20%.
- Sen2Cor: Software that performs atmospheric, terrain, and cirrus correction to SEN2 Level-1C input data^12^. We store the Scene Classification (SC), which provides a semantic pixel-level classification map. The SC maps are obtained from the “COPERNICUS/S2_SR” GEE dataset.
- s2cloudless: Single-scene CD algorithm created by Sentinel-Hub using a LightGBM decision tree model^50^. The cloud probability values are collected without applying neither a threshold nor dilation. This resource is available in the “COPERNICUS/S2_CLOUD_PROBABILITY” GEE dataset.
- CD-FCNN: U-Net with two different SEN2 band combinations:^23^ RGBI (B2, B3, B4, and B8) and RGBISWIR (B2, B3, B4, B8, B11, and B12) trained on the Landsat Biome-8 dataset (transfer learning^52,53^ from Landsat 8 to Sentinel-2).
- KappaMask: U-Net with two distinct settings:^54^ all Sentinel-2 L1C bands and all Sentinel-2 L2A bands except the Red Edge 3 band. It was trained using both Sentinel-2 KappaZeta Cloud and Cloud Shadow Masks and the Sentinel-2 Cloud Mask Catalogue (see Table 1).
- QA60: Cloud mask embedded in the quality assurance band of SEN2 Level-1C products.

Table 5 shows the cloud semantic categories for the different CD techniques available in CloudSEN12. It should be noted that only four CD algorithms provide the cloud shadow category.

**Table 5:** Output correspondence for the available CD algorithms.

CloudSEN12	KappaMask	Sen2Cor	Fmask	s2cloudless	CD-FCNN	QA60
0 Clear	1 Clear	4 Vegetation	0 Clear land	0 Clear	0 Clear	0 Clear
		2 Dark area pixels	1 Clear water			
		5 Bare Soils	3 Snow			
		6 Water				
		11 Snow				
1 Thick cloud	4 Cloud	8 Cloud medium probability	4 Cloud	1 Cloud	1 Cloud	1024 Opaque cloud
		9 Cloud high probability				
2 Thin cloud	3 Semi-transparent cloud	10 Thin cirrus				2048 Cirrus cloud
3 Cloud shadow	2 Cloud shadow	3 Cloud shadows	2 Cloud shadow			

KappaMask, Sen2Cor, Fmask, S2cloudless, CD-FCNN and QA60 are mapped respectively to CloudSEN12 semantic categories. Adapted from Sanchez *et al*.^13^.

### Preparing CloudSEN12 for machine learning

**Fig. 8:**
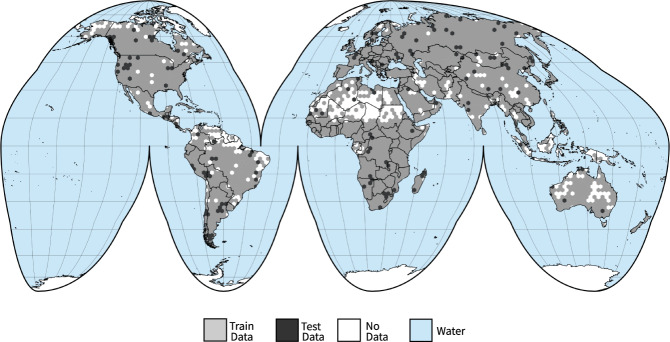
Location of the training (grey) and testing (black) regions. The IPs were collocated in a equal-area hexagonal discrete grid with a facet size of 140 km.

Splitting our densely annotated dataset into train and test sets is critical to ensure that ML practitioners always use the same samples when providing results. Since cloud formation tends to fluctuate smoothly throughout space, a simple random split is suspicious to violate the assumption of test independence, especially under highly clustered labeled areas, such as the green and yellow regions shown in Fig. 1. Therefore, we carry out a spatially stratified block split strategy^55^, based on Roberts *et al*.^56^, to limit the risk of overfitting induced by spatial autocorrelation. First, we divided the Earth’s surface into regular hexagons of 50,000 *km*^2^. Then, the initial hexagons are filtered, retaining only those intersecting with the high-quality subset. Finally, using the difficulty IP property (see Table 3), we randomly stratified the remained hexagon blocks using 90% (1827 ROIs) and 10% (173 ROIs) for training and testing, respectively (Fig. 8). Notice that on each ROI, we have five IPs hence the total amount of training and testing data is five times these numbers. The no annotation and scribble subsets might be used as additional inputs for the training phase.

## Data Records

The dataset is available online via Science Data Bank^57^. We defined an IP as the primary atomic unit, representing a single spatio-temporal component. Each IP has 49 assets (see Table 2) and 31 properties (see Table 4). All the assets are delivered in the form of LZW-compressed COG (Cloud Optimized GeoTIFF) files. COG is a standard imagery format for web-optimized access to raster data. It has a specific internal pixel structure that allows clients to request just specified areas of a large image by submitting HTTP range requests^58^. The IP properties are shared using the SpatioTemporal Asset Catalog (STAC) specification. STAC provides a straightforward architecture for reading metadata and assets in JSON format, providing users with a sophisticated browsing experience seamlessly integrating with modern scripting languages and front-end web technologies.

Figure 10 shows the CloudSEN12 dataset organization, which follows a four-level directory structure. The top level includes the metadata file in CSV format (content of Table 4) and three folders: high, scribble, and no-label. These folders correspond to the annotation categories: high-quality (2000 ROIs), scribble (2000 ROIs), and no annotation (5880 ROIs). The second-level folders correspond to each specific geographic location (ROI). The folder name is the ROI ID (Fig. 10b). Since an ROI consists of five IPs with different cloud coverage, each ROI folder contains five folders whose names match the GEE Sentinel-2 product ID of the specific IP (Fig. 10c). Finally, each IP folder stores the assets detailed in Table 2 (Fig. 10d).

## Technical Validation

### Neural network architecture

In order to demonstrate cloudSEN12’s effectiveness in developing DD models, we trained a U-Net^59^ network with a MobileNetV2^60^ backbone (UNetMobV2) using only the high-quality pixel-level annotation set. U-Net models often have considerable memory requirements since the encoder and decoder components include skip connections of large tensors. However, the MobileNetV2 encoder significantly decreases memory utilization due to the use of depthwise separable convolutions and inverted residuals. The entire memory requirements of our model, considering a batch with a single image (1 × 13 × 512 × 512), the forward/backward pass, and model parameters, is less than 1 GB using the PyTorch deep learning library^61^. The implementation of the proposed model can be found at https://github.com/cloudsen12/models.

The high-quality set is split into training, validation, and test sets. First, we obtain the test and no-test set using the previous geographical blocks. Then, the no-test set is randomly divided into training and validation sets according to the ratio of 90/10%. The U-Net network is trained considering all the SEN2 L1C bands with a batch size of 32, Adam optimizer with a learning rate of 10^−3^, and the standard cross-entropy as loss function. During the training phase, the learning rate is lowered by a factor of 0.10 if the cross-entropy measured in the validation set does not improve in four epochs. Lastly, if the model does not improve after ten epochs, the model with the lowest cross-entropy value in the validation set is chosen.

### Benckmarking strategy

CloudSEN12’s suitability for benchmarking cloud and cloud shadow is discussed in this section. In order to maintain fairness, we only consider the 975 IPs available in the test set. We assessed the similarity between the semantic categories (Table 3) from CD models (automatic) and manual annotations through three experiments. First, we created the “cloud” and “non-cloud” superclasses (Table 3) that aggregate thick and thin cloud and clear and cloud shadows classes, respectively. In the second experiment, cloud shadows are validated by considering four algorithms: UNetMobV2, KappaMask, Fmask, and Sen2Cor, as not all algorithms are capable of detecting cloud shadows (Table 5). Finally, in the third experiment, “valid” and “invalid” superclasses (Table 3) are also analyzed just for algorithms with cloud shadow detection. In all the experiments, human-level performance is included by comparing manual annotations before and after the quality control procedure (see Method: Quality control phase section). We report producer’s accuracy (PA), user’s accuracy (UA), and balanced overall accuracy (BOA) as metrics to assess the disparities between predicted and expected pixels:

1 $${\rm{PA}}=\frac{TP}{TP+FN}\quad \quad {\rm{UA}}=\frac{TP}{TP+FP}\quad \quad {\rm{BOA}}=0.5\left(PA+\frac{TN}{TN+FP}\right)$$

Where *TP*, *TN*, *FP*, and *FN* denote true positive, true negative, false positive, and false negative. High PA values show that cloud pixels have been effectively masked out (clear-sky conservative approaches). In contrast, high UA values indicate that the algorithm is cautious in excluding non-cloud pixels (conservative cloud approaches). High BOA values are related to a good balance of false positives and false negatives. We generate a unique set of PA, UA, and BOA values for each test IP. Since the PA and UA values are always zero in cloudless IPs, they were replaced by NaN to prevent negative bias in the results. Then to report the summarized PA and UA metrics (Table 6), we consider the following three scenarios: (i) low values group (*PA*_*low*_% and *UA*_*low*_%), which represents the percentage of IPs with PA/UA values lower than 0.1; (ii) middle values group (*PA*_*middle*_% and *UA*_*middle*_%) which represents the percentage of IPs between 0.1 and 0.9; (iii) high values group (*PA*_*high*_% and *UA*_*high*_%) which represents the percentage of total IPs higher than 0.9. In contrast to UA and PA, we calculate the median of all IPs for BOA estimates.

**Table 6:** Metrics of the three different experiments for all the annotation algorithms.

Experiment	CD algorithm	BOA	PA_*low*_%	PA_*middle*_%	PA_*high*_%	UA_*low*_%	UA_*middle*_%	UA_*high*_%
**a. cloud/no cloud**	**Human level**	0.99	1.03	14.03	84.94	0.13	4.39	95.48
	**UNetMobV2**	**0.92**	**0.77**	**30.63**	**68.6**	0.26	25.03	74.71
	**KappaMask L2A**	0.77	2.83	31.92	65.25	1.56	63.04	35.41
	**KappaMask L1C**	0.82	4.89	45.3	49.81	0.65	38.38	60.97
	**Fmask**	0.84	5.92	40.54	53.54	0.26	52.65	47.09
	**s2cloudless**	0.79	7.08	52.38	40.54	0.65	31.5	67.84
	**Sen2Cor**	0.71	13.13	64.86	22.01	1.58	20.05	78.36
	**QA60**	0.58	24.84	49.94	25.23	1.39	37.62	60.99
	**CD-FCNN-RGBI**	0.72	17.50	74.00	8.49	1.62	12.58	85.79
	**CD-FCNN-RGBISWIR**	0.72	18.40	71.43	10.17	**0.82**	**9.43**	**89.75**
**b. cloud shadow**	**Human level**	0.99	3.11	22.04	74.85	0.60	9.97	89.43
	**UNetMobV2**	**0.89**	**8.88**	**67.16**	**23.96**	7.99	46.65	45.36
	**KappaMask L2A**	0.64	37.28	59.76	2.96	12.24	36.9	50.85
	**KappaMask L1C**	0.74	30.03	60.95	9.02	20.67	59.36	19.97
	**Fmask**	0.72	22.34	76.04	1.63	14.53	77.06	8.41
	**Sen2Cor**	0.51	64.5	35.21	0.30	**6.90**	**18.10**	**75.00**
**c. valid/invalid**	**Human level**	0.99	1.03	14.8	84.17	0.13	2.33	97.55
	**UNetMobV2**	**0.91**	**0.77**	**28.57**	**70.66**	**0.00**	**17.14**	**82.86**
	**KappaMask L2A**	0.75	2.96	39.38	57.66	1.29	44.32	54.39
	**KappaMask L1C**	0.81	3.99	47.62	48.39	0.65	32.64	66.71
	**Fmask**	0.81	4.89	45.43	49.68	0.26	44.34	55.39
	**Sen2Cor**	0.67	13.77	69.63	16.6	1.05	18.58	80.37

The BOA value is computed as the median BOA across all IPs. PA/UA values show the percentage of IPs with that metric below 0.1 (low), between 0.1 to 0.9 (middle), and higher than 0.9 (high). Values closest to one hundred in the “high” group are better, whereas values close to zero in the other two groups are the ideal. The best values considering the PA/UA high group have been highlighted in bold (excluding human annotation).

### Cloud vs non-cloud

**Fig. 9:**
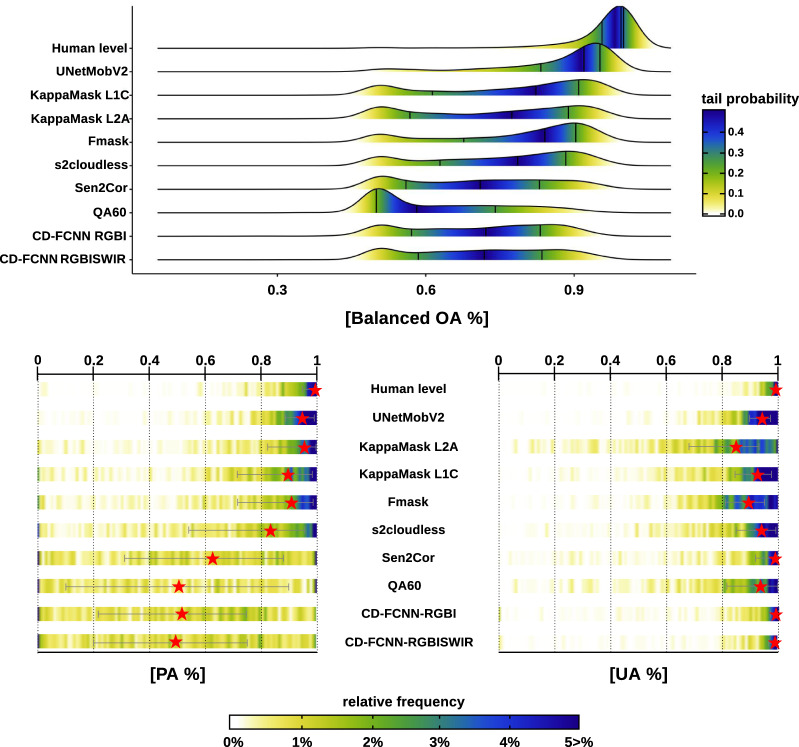
BOA, PA, and UA comparison for the CloudSEN12 dataset. The upper figure depicts BOA density estimations for all CloudSEN12 IPs high-quality. The colors reflect the tail probability estimated by 0.5-*abs*(0.5-*ecdf*), where *ecdf* is the empirical cumulative distribution function. The vertical black lines drawn represent the first, second, and third quartiles, respectively. The heatlines in the lower figure shows the PA and UA value distribution. The red stars shows the median and the gray lines the 25th and 75th percentiles.

Figure 9 and Table 6a show BOA, PA, and UA density error curves and summary statistics for the first experiment. Excluding UNetMobV2 results, BOA and PA values exhibited a well-defined binomial error distribution with peak modes of different intensities. We found that the mode of the secondary peak is close to 0.5 and 0 for BOA and PA, respectively. Considering the three algorithms with the highest BOA, we found that this secondary distribution contains at least 3.86% of the total IPs (see PA_*low*_ in Table 6a) and 38.83% of the IPs fall between the transition of these two distributions (see PA_*middle*_ in Table 6a). A simple visual examination reveals that the omission of small and thin clouds is the primary cause of PA_*low*_ values, whereas PA_*middle*_ is mainly attributable to cloud borders misinterpretation. Low-thickness clouds, such as cirrus and haze, tend to produce more omission errors independent of the cloud detection algorithm. In KD algorithms, this can be explained by the simplicity of semitransparent cloud modules, which are just a conservative threshold in the cirrus band (B10). Additionally, thin clouds are often overlooked or unfairly reported in most CD datasets^62^. In the primary distribution, the peak’s mode is close to 0.90 and 0.95 for BOA and PA values, holding 57.31% of the IPs (see PA_*high*_ in Table 6a). These results suggest that more than half of the IPs in CloudSEN12 are easily recognizable by automatic cloud masking algorithms.

**Fig. 10:**
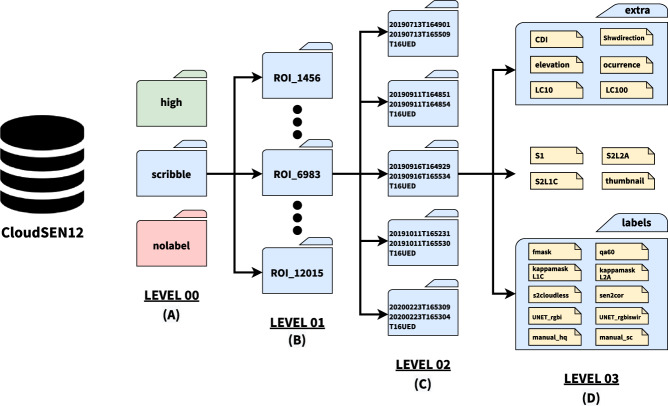
Folder structure of the CloudSEN12 dataset. level zero: type of manual annotation, level one: geographic location (ROI), level 2: IP (for each ROI there are 5 IPs with different amount of cloud coverage), level 3: asset level where files in COG formats are stored.

Figure 9 demonstrates furthermore that not all algorithms exhibit the same behavior. Based on the PA and UA metrics, we may differentiate between three types of algorithms: quite balanced (UNetMobV2, Fmask, and KappaMask L1C), cloud conservative (CD-FCNN, QA60, s2cloudless, and Sen2Cor), and non-cloud conservative (KappaMask L2A). The first group reports similar values between PA_*high*_ and UA_*high*_ percentages. In contrast, the second group exhibits high UA values at the expense of worsening PA. As observed in the PA heatline plot, these algorithms show a pronounced bimodal distribution and a wide interquartile range, with more than half of the IPs exhibiting PA values below 0.5. Considering the high temporal resolution of SEN2 imagery, it seems unsuitable to use cloud-conservative algorithms for CD, except maybe for extremely cloudy regions where each clear pixel is critical^62^. On the other hand, in non-cloud conservative algorithms, over half of all IPs have PA values greater than 0.9 (see column PA_*high*_ in Table 6a), but as a result, the UA_*high*_ metric decrease significantly.

Based on BOA estimates (see column BOA in Table 6a), we may conclude that QA60 is the most unreliable algorithm, failing to distinguish both cloud and non-cloud pixels. Whereas UNetMobV2 is clearly the best at detecting clouds, even semitransparent and small clouds, that other algorithms usually overlook. Although the UNetMobV2 and KappaMask are based on a similar network, we observe that KappaMask (in particular version 2A) tends to overestimate clouds under specific land cover types, such as mountains, open/enclosed water bodies, and coastal environments. Considering that the L1C and L2A versions of KappaMask are fine-tuned on a relatively small dataset from Northern Europe, it is expected that fine-tuning in CloudSEN12 should lead to better results on a global evaluation. Finally, we can conclude that UNetMobV2, Fmask, and KappaMask level 1C provide the most stable solution for cloud masking, with inaccuracies evenly distributed across different cloud types and land covers.

### Cloud shadows

Quantitative evaluations of cloud shadow detection on CloudSEN12 are presented in Table 6b. The percentage of IPs with PA values < 0.1 (PA_*low*_) ranges from 64.50% for Sen2Cor to 8.88% for UNetMobV2, indicating that a large number of cloud shadow pixels are omitted in all the algorithms. In contrast to the cloud/no-cloud experiment, the vast majority of IPs belong to the PA_*middle*_ and UA_*middle*_ groups, except for Sen2Cor, which belongs to the PA_*low*_ group. The PA_*high*_ percentage value was unexpectedly low, suggesting that the ground truth and predicted values rarely collocate perfectly over the same area. Comparing the results of the first and second experiments reveals that correctly detecting thick and thin clouds is not guaranteed for achieving a high PA score in cloud shadows. Besides, our results suggest that DL-based approaches (KappaMask and UNetMobV2) outperform KD algorithms (Sen2Cor and Fmask). This seems reasonable, given that KappaMask and UNetMobV2 are built on a multi-resolution model. Hence, it is probable that the model learns to identify the spatial coherence between clouds and cloud shadows classes.

### Valid vs invalid

In this section, we examine the combined detection of cloud and cloud shadows of five automatic CD algorithms (see Table 6c). The reported metrics show a slight decrease in the PA_*high*_ values of Fmask, Sen2Cor, and KappaMask L1C models compared to the first experiment. Consequently, the KappaMask L2A model significantly lowers its PA_*high*_ value from 65.25 to 57.66%, indicating that this model tends to confuse cloud shadow with clear pixels. In contrast, UNetMobV2 slightly increased its reported PA_*high*_ value from 68.60 to 70.66%. This is explained by the fact that UNetMobV2 tends to err thin cloud pixels with cloud shadows and vice versa, and since both belong to the same superclass in this experiment, these inconsistencies are considered true positives. Finally, further studies are required to identify the circumstances in which CD algorithms depart most from human-level performance to deliver superior automatic CD algorithms.

### Discussion of experimental results

In the three experiments, UNetMobV2 delivers the best balance between false negative and false positive errors. These outcomes are expected due to the more extensive and diverse image patches utilized during training. However, because deep learning models are prone to handle target shift poorly, the use of other datasets (e.g., PixBox^63^ or Hollstein^28^) might aid in corroborating these findings. Furthermore, the Sen2Cor results are estimated without considering changes between different versions (Supplementary Fig. S3). Therefore, the values reported here could vary from those obtained using only the latest version (version 2.10, accessed on 9 July 2022). In addition, it is important to note that, in contrast to FMask and Sen2Cor, KappaMask and UNetMobV2 results are produced without image boundary data. Therefore, expanding the IP size might improve the reported metrics, particularly for the cloud shadows experiment.

## Usage Notes

This paper introduces CloudSEN12, a new large dataset for cloud semantic understanding, comprising 49,400 image patches distributed across all continents except Antarctica. The dataset has a total size of up to 1 TB. Nevertheless, we assume most user experiments need only a fraction of CloudSEN12. Therefore, to simplify its use, we developed a Python package called *cloudsen12* (https://github.com/cloudsen12/cloudsen12). This Python package aims to help machine learning and remote sensing practitioners to:

- Query and download cloudSEN12 using a user-friendly interface.
- Predict cloud semantics using the trained UNetMobV2 model.

The CloudSEN12 website https://cloudsen12.github.io/ includes tutorials for querying and downloading the dataset using the *cloudsen12* package. Besides, there are examples of how to train DL models using PyTorch. Finally, although CloudSEN12 was initially designed for cloud semantic segmentation, it can be easily adapted to tackle other remote sensing problems like SAR-sharpening^64^, colorizing SAR images^65^, and SAR-optical image matching^66^. Furthermore, by combining CloudSEN12 with ESA WorldCover 10 m v100, users may train land cover models to be aware of cloud contamination.

## Supplementary information

Supplementary Figures

## Data Availability

The code to (1) create the raw CloudSEN12 imagery dataset, (2) download assets associated with each ROI, (3) create the manual annotations, (4) build and deploy cloudApp, (5) generate automatic cloud masking, (6) reproduce all the figures, (7) replicate the technical validation, (8) modify *cloudsen12* Python package, and (9) train DL models are available in our GitHub organization https://github.com/cloudsen12/.
