# Supplementary material for: CloudSEN12, a global dataset for semantic understanding of cloud and cloud shadow in Sentinel-2
Source: Sci Data. 2022 Dec 24;9:782. doi: 10.1038/s41597-022-01878-2 (PMC9789947; doi:10.1038/s41597-022-01878-2)
Supplement: Supplementary file 1 — Supplementary Figures [file 41597_2022_1878_MOESM1_ESM.pdf]

Figure S1	IRIS (Intelligently Reinforced Image Segmentation) graphical user interface. There are seven feature bars. A) Edit and navigation bar. B) Select drawing semantic classes. C) Draw bar; the last bottom of this group executes the GBDT algorithm that fills out the mask using prior manual annotations. D) Testing bar, it helps to compare human and AI annotations. E) Image contrast bar that changes image brightness and saturation. F) Image metadata that displays image thumbnail and IP location using Google maps. G) Machine learning summary support that shows GBDT performance metrics. The IRIS interface displays the Cirrus band, Red-Green-Blue and Blue-SWIR1-SWIR2.
Figure S2	Three main cloudApp panels. A) Display time series for the Blue, SWIR1 bands, and NDVI for all images in a one-year moving window with less than 5\% cloud coverage. B) Inspect image thumbnails; the white circle's values are averaged and displayed in panel A. C) Map display for showing the image patch's centroid.
Figure S3	Different versions of Sen2Cor in the test set.

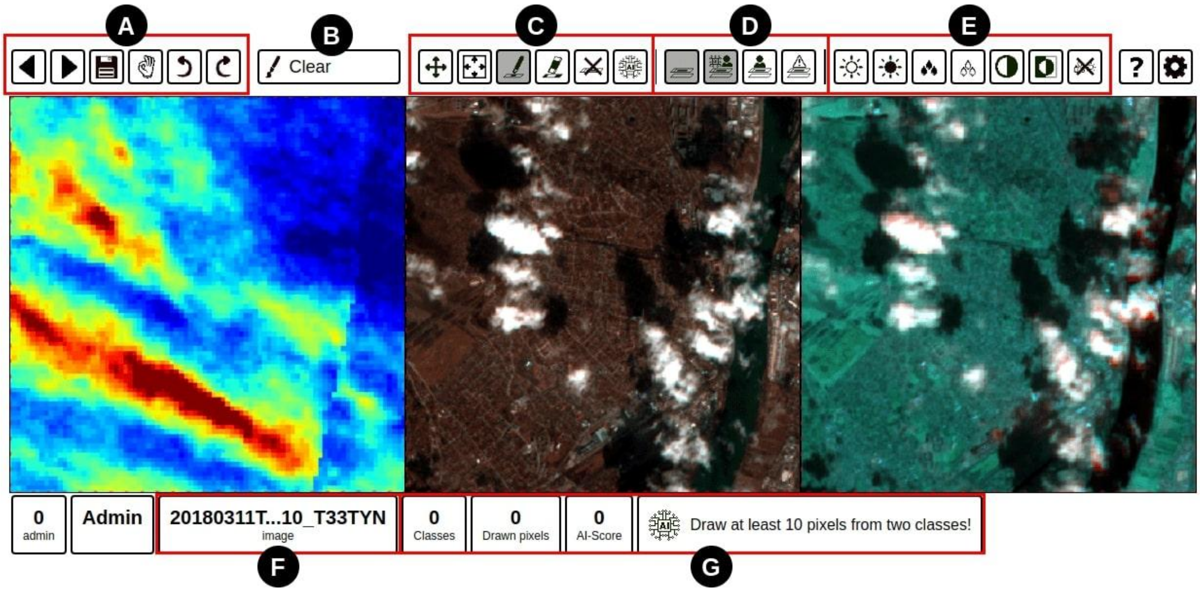

Figure S1: IRIS (Intelligently Reinforced Image Segmentation) graphical user interface. There are seven feature bars. A) Edit and navigation bar. B) Select drawing semantic classes. C) Draw bar; the last bottom of this group executes the GBDT algorithm that fills out the mask using prior manual annotations. D) Testing bar, it helps to compare human and AI annotations. E) Image contrast bar that changes image brightness and saturation. F) Image metadata that displays image thumbnail and IP location using Google maps. G) Machine learning summary support that shows GBDT performance metrics. The IRIS interface displays the Cirrus band, Red-Green-Blue and Blue-SWIR1-SWIR2.

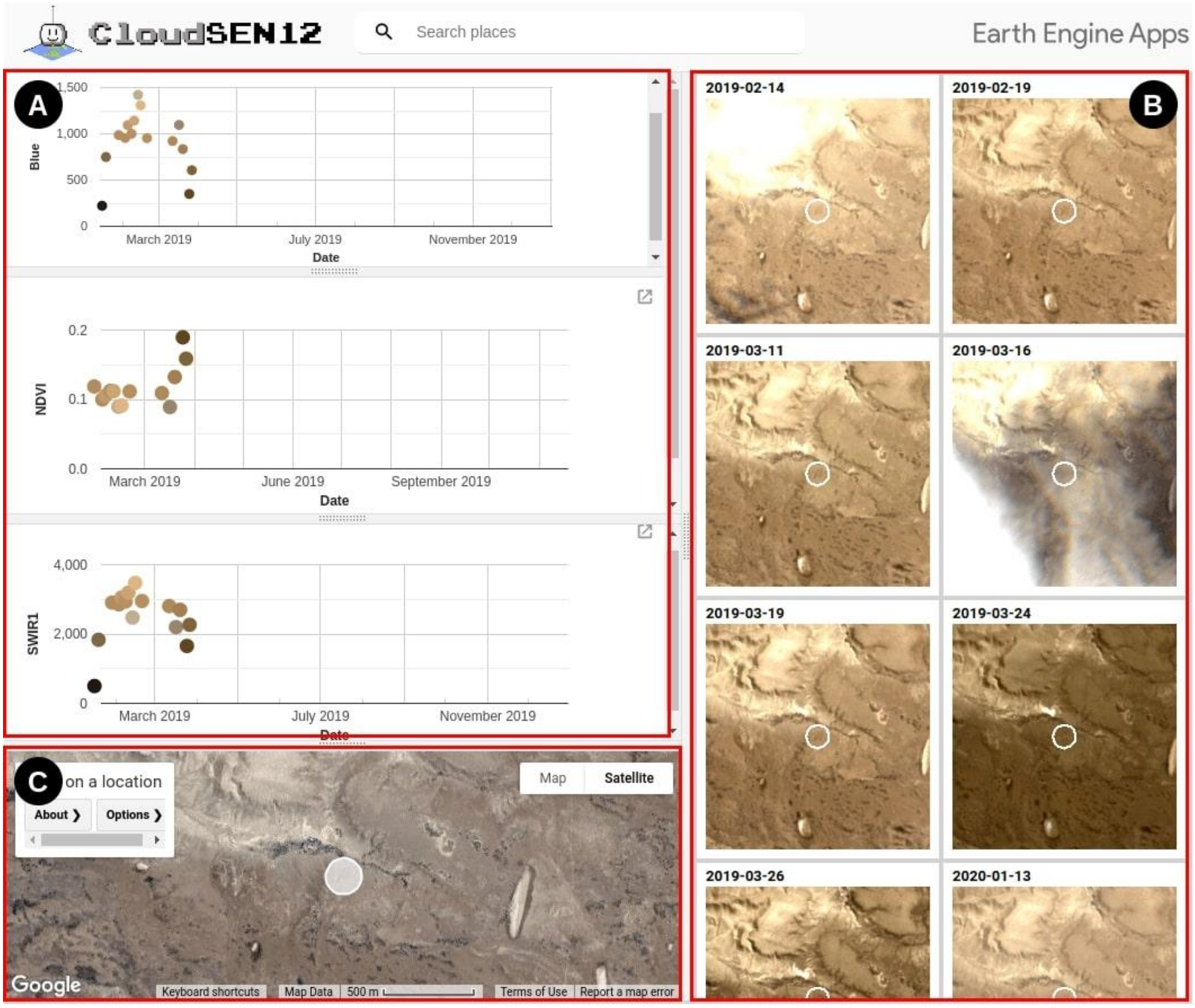

Figure S2: Three main cloudApp panels. A) Display time series for the Blue, SWIR1 bands, and NDVI for all images in a one-year moving window with less than 5\% cloud coverage. B) Inspect image thumbnails; the white circle's values are averaged and displayed in panel A. C) Map display for showing the image patch's centroid.

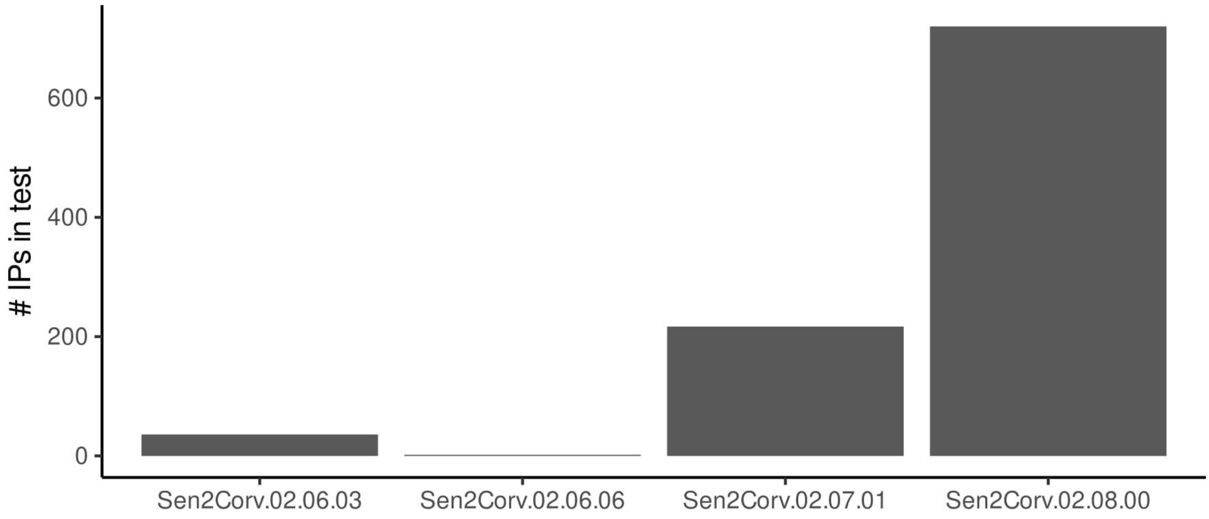

Figure S3: Different versions of Sen2Cor in the test set.
